# Draft Genome Sequences of Two Hydrogenogenic Carboxydotrophic Bacteria, *Carboxydocella* sp. Strains JDF658 and ULO1, Isolated from Two Distinct Volcanic Fronts in Japan

**DOI:** 10.1128/genomeA.00242-17

**Published:** 2017-04-20

**Authors:** Yuto Fukuyama, Tatsuki Oguro, Kimiho Omae, Yasuko Yoneda, Takashi Yoshida, Yoshihiko Sako

**Affiliations:** Laboratory of Marine Microbiology, Graduate School of Agriculture, Kyoto University, Kyoto, Japan

## Abstract

Hydrogenogenic carboxydotrophs may provide hydrogen as primary energy for the microbial community via carbon monoxide oxidation. To investigate the genetics of carbon monoxide metabolism, we report here the draft genome sequences of the hydrogenogenic carboxydotrophs *Carboxydocella* sp. strains JDF658 (2.60 Mbp; G+C content, 49.2%) and ULO1 (2.70 Mbp; G+C content, 48.8%).

## GENOME ANNOUNCEMENT

Carbon monoxide (CO), a gas that is toxic to many organisms, originates from both biological and abiological sources in volcanic environments (1). As anaerobic, thermophilic, and hydrogenogenic carboxydotrophs, *Carboxydocella* spp. can utilize CO for their growth, producing hydrogen, which can be an energy source for the microbial community in volcanic environments (2, 3). However, limited genomic information is available for the genus *Carboxydocella*. We isolated two novel strains of *Carboxydocella*, JDF658 and ULO1, from a deposit of an open-air stream from a hot spring well and the sediment of a maar lake bordering the volcanic fronts of Izu-Bonin Trench and Ryukyu Trench in Japan, respectively (4). Here, we report the draft genome sequences of the two *Carboxydocella* species.

Genomic libraries were prepared from purified DNA of strains JDF658 and ULO1 using a Nextera XT DNA sample prep kit (Illumina, Inc., San Diego, CA, USA), followed by sequencing with the Illumina MiSeq platform using a version 2 reagent kit (2 × 150-bp paired-end reads). A total of 1,619,582 and 4,679,334 paired-end reads were generated for strains JDF658 and ULO1, respectively. High-quality reads (Phred score > Q30 for 80% of bases) were assembled into contigs using Velvet 1.2.10 (5). The assembled contigs were subjected to the Microbial Genome Annotation Pipeline (http://www.migap.org/index.php/en) (6) to predict open reading frames (ORFs), followed by manual curation. Subsequently, protein sequences were annotated using BLASTp searches (7, 8) against nonredundant protein sequences available in the National Center for Biotechnology Information database (9).

The draft genomes of strains JDF658 and ULO1 were assembled into 270 and 180 contigs with total lengths of approximately 2.60 Mbp and 2.70 Mbp, respectively. These draft genomes showed average G+C contents of 49.2% and 48.8%, containing 2,731 and 2,804 predicted ORFs, respectively.

Carboxydotrophs possess at least one CO dehydrogenase (CODH) for interconversion between CO and CO_2_ (10). We identified CODH genes (*cooS*) in the two novel *Carboxydocella* strains and compared their genomic contexts with those of their relatives and other carboxydotrophs to predict CODH function (11). In both strains, we identified three distinct *cooS* genes that encoded conserved amino acid sequences in active centers (12, 13). The genomic contexts of the three *cooS* genes differed from each other but were well conserved to counterparts among our strains and *Carboxydocella sporoproducens* (IMG genome identification [ID] 2568526009) that were isolated from geologically distinct volcanic regions. Two of the three *cooS* genes were identified in a well-characterized genomic context: an energy-converting hydrogenase gene cluster and an acetyl-coenzyme A (acetyl-CoA) synthetase gene cluster. Complexes of these gene products are considered to be responsible for energy conversion by hydrogen production via hydrogenogenic CO metabolism and carbon fixation via the Wood-Ljungdahl pathway, respectively (11, 14). The final *cooS* gene was identified in a genomic context of unknown function. This CODH was phylogenetically distinct from well-characterized CODHs, branching deeply from a clade including still-less-understood CODHs that lack some conserved amino acids in active centers (15). The third CODH is potentially active, and physiological study could reveal its function in the CO metabolism of *Carboxydocella* species.

### Accession number(s).

The draft genome sequences of *Carboxydocella* species strains JDF658 and ULO1 have been deposited in the DNA Data Bank of Japan under the GenBank accession numbers BDLR01000000 and BDLQ01000000, respectively.
